# An integrated drug repurposing strategy for the rapid identification of potential SARS-CoV-2 viral inhibitors

**DOI:** 10.1038/s41598-020-70863-9

**Published:** 2020-08-17

**Authors:** Alfonso Trezza, Daniele Iovinelli, Annalisa Santucci, Filippo Prischi, Ottavia Spiga

**Affiliations:** 1 Department of Biotechnology, Chemistry and Pharmacy, University of Siena, 53100 Siena, Italy; 2 School of Life Sciences, University of Essex, Colchester, CO4 3SQ UK

**Keywords:** Biochemistry, Computational biology and bioinformatics, Drug discovery, Structural biology

## Abstract

The Coronavirus disease 2019 (COVID-19) is an infectious disease caused by the severe acute respiratory syndrome-coronavirus 2 (SARS-CoV-2). The virus has rapidly spread in humans, causing the ongoing Coronavirus pandemic. Recent studies have shown that, similarly to SARS-CoV, SARS-CoV-2 utilises the Spike glycoprotein on the envelope to recognise and bind the human receptor ACE2. This event initiates the fusion of viral and host cell membranes and then the viral entry into the host cell. Despite several ongoing clinical studies, there are currently no approved vaccines or drugs that specifically target SARS-CoV-2. Until an effective vaccine is available, repurposing FDA approved drugs could significantly shorten the time and reduce the cost compared to de novo drug discovery. In this study we attempted to overcome the limitation of in silico virtual screening by applying a robust in silico drug repurposing strategy. We combined and integrated docking simulations, with molecular dynamics (MD), Supervised MD (SuMD) and Steered MD (SMD) simulations to identify a Spike protein – ACE2 interaction inhibitor. Our data showed that Simeprevir and Lumacaftor bind the receptor-binding domain of the Spike protein with high affinity and prevent ACE2 interaction.

## Introduction

The World Health Organisation (WHO) declared the Coronavirus disease (COVID-19) outbreak a pandemic on March 12th 2020, and as of May 21st, over 4,893,186 cases and 323,256 deaths have been reported (https://www.who.int/emergencies/diseases/novel-coronavirus-2019/situation-reports/). The Severe acute respiratory syndrome–coronavirus 2 (SARS-CoV-2) was identified as the viral agent causing the disease. SARS-CoV-2 is closely related to the SARS-CoV, which caused a pandemic in 2002–2003^1^, and it is believed to be the third member of the *Coronaviridae* family to cause severe respiratory diseases in human^2^. Despite several ongoing clinical studies, there are currently no approved vaccines or drugs that specifically target SARS-CoV-2.

SARS-CoV-2 has a single-stranded positive-sense RNA composed of 29,903 nt containing five genes, *ORF1ab* (codifying 16 non-structural proteins), *spike* (S), *envelope* (E), *membrane* (M) and *nucleocapsid* (N) genes^3^. The virus uses the S homotrimeric glycoprotein located on the virion surface to allow entry into the human cells^4^. The S protein goes through major structural rearrangements to mediate viral and human cell membranes fusion. The process is initiated by the binding of the receptor-binding domain (RBD) of the S1 subunit to the peptidase domain (PD) of angiotensin-converting enzyme 2 receptor (ACE2) on the host cell^5^. Structural studies have shown that two S protein trimers can simultaneously bind to one ACE2 dimer^6^. This induces a conformational change that expose a proteolytic site on the S protein, which is cleaved by the cellular serine protease TMPRSS2^7^. Dissociation of S1 induces transition of the S2 subunit to a post fusion conformation, with exposed fusion peptides^8^, which allows endocytic entry of virus^9^. Wrapp et al.^10^ have shown that, despite SARS-CoV-2 and SARS-CoV sharing a similar cell entry mechanism, SARS-CoV-2 S protein binds ACE2 with a 10- to 20-fold higher affinity than SARS-CoV S, which may be related to the higher person-to-person transmission of SARS-CoV-2.

S glycoprotein is highly immunogenic, and it is a promising target for drug design^11^. We showed that a combination of four 20-mer synthetic peptides disrupting SARS-CoV S heterotrimer reduced or completely inhibited infectivity in vitro^12^. Similarly, antibodies targeting SARS-CoV S protein neutralize the virus and have potential for therapy^13^. In fact, disruption of the binding of the S protein to ACE2 prevents the virus from attaching to the host cell^14^.

The social and economic impact of COVID-19 and the possibility of future similar pandemics are pushing for the rapid development of treatments. As such, targeting viral-host protein–protein interaction (PPI) may represent a promising way to prevent or reduce the spreading of the virus before a vaccine is available^15^. In this study we performed an extensive analysis of the intrinsic dynamic, structural properties and drug targeting of SARS-CoV-2 RDB. In particular starting from the structure of RDB in complex with ACE2, we identified transient pockets on RDB on the ACE2 interaction surface area. Our data provide detailed information on the dynamic features of RDB that we exploited for docking studies. We carried out a virtual screening using 1582 FDA-approved drugs to explore new therapeutic benefits of existing drugs. To take into account unique features of molecules, such as conformational flexibility, charges distribution, and solvent role in target recognition and binding, we performed an extensive molecular dynamics simulation analysis. By combining molecular dynamics simulations (MD), Supervised MD (SuMD), Steered MD (SMD) and interaction energy calculations, we showed that Simeprevir and Lumacaftor bind RDB with high affinity and prevent ACE2 interaction. Overall, by adopting a robust in silico approach, our results could open the gates toward the development of novel COVID-19 treatments.

## Methods

### Structural resources

3D Structure and FASTA sequence of SARS-CoV-2 RBD in complex with human hACE2 (PDB ID 6LZG) were retrieved from the RCSB Protein Data Bank^16^. To avoid errors during the molecular dynamic (MD) simulations, missing side chains and steric clashes in PDB files were adjusted through homology modelling, using PyMOD2.0 and MODELLER v.9.3^17^. 3D structures were validated using PROCHECK^18^. GROMACS 2019.3^19^ with charmm36-mar2019 force field was used to resolve high energy intramolecular interaction before docking simulations, and CGenFF was used to assign all parameters to ligands. Structures were immersed in a cubic box filled with TIP3P water molecules and counter ions to balance the net charge of the system. Simulations were run applying periodic boundary conditions. The energy of the system was minimized with 5.000 steps of minimization with the steepest descent algorithm and found to converge to a minimum energy with forces less than 100 kJ/mol/nm. A short 10 ns classic Molecular Dynamics (cMD) was performed to relax the system.

All the cMD simulations were performed integrating each time step of 2 fs; a V-rescale thermostat maintained the temperature at 310 K and Berendsen barostat maintained the system pressure at 1 atm, with a low dumping of 1 ps^−1^; the LINCS algorithm constrained the bond lengths involving hydrogen atoms.

### Transient pockets and virtual screening

A 100 ns cMD simulation was used, as described above, for the identification of transient pockets. Transient pockets were identified by analysing MD trajectories of SARS-CoV-2 RBD structure with EPOS tool^20^, using parameters by default. The volumes of the transient pockets during the simulation were measured using POVME^21^. Open pockets in close proximity to ACE2 binding site were selected based on the depth and polarity of the cavity. A box with dimensions of 25, 35, and 20 Å was created around the transient pocket using Autodock Tools^22^. Subsequently, a virtual screening using the FDA-approved drug library available on DrugBank^23^ was carried out on SARS-CoV-2 RBD using AutoDock/VinaXB^24^. MGLTOOLS scripts^22^ and OpenBabel^25^ were used to respectively convert protein and ligand files and to add gasteiger partial charges. The full FDA-approved library was downloaded from DrugBank^23^ and a total of 1,582 molecules, corresponding to the number of structures successfully processed by OpenBabel, was used for the virtual screening. Drugs names and structures used in this study are available on https://github.com/fprischi/Supplementary_FDAlibrary.git

### Supervised molecular dynamics (SuMD) simulations

SuMD were used to sample the binding of hACE2 to RBD and to probe the binding of hACE2 to RBD-Simeprevir/Lumacaftor complexes. SuMD methodology relies on a tabu-like algorithm that monitors the distance between hACE2 and centre of mass of the RBD binding site during unbiased MD simulations to sample a binding event in the range of nanoseconds^26^. The protocol is based on performing a series of short unbiased MD simulations, where after each simulation the distance points collected at regular time intervals are fitted into a linear function. If the resulting slope is negative, then hACE2 is getting closer to the RBD binding site and the MD steps are kept. If the slope is not negative, then the simulation is restarted by randomly assigning the atomic velocities. We used an SuMD step of 1,000 ps with a constant temperature and pressure of 310 K and 1 atm respectively. When the distance between the hACE2 and RBD reached 5 Å or less, then the supervision was disabled and a 10 ns cMD simulation was performed. The analysis was performed with an in-house written python and bash script.

### Steered molecular dynamics (SMD) simulations

In order to evaluate the binding interaction between RBD and Simeprevir or Lumacaftor, the RBD-Simeprevir/Lumacaftor complexes were simulated to dissociate using a 700 ps SMD simulation by Constant Force Pulling of 250 kJ/mol/nm. While the backbone of RBD was not allowed to move, Simeprevir and Lumacaftor experienced a constant force in x, y, z direction, specifically (250, 0, 0) for both compounds. Simeprevir and Lumacaftor were pulled with an external force in the NPT ensemble at 1 atm and 310 K with 2 fs time steps. MD analyses was performed with GROMACS 2019.3 package and displayed with GRACE.

### Protein–ligand interaction energy

To quantify the strength of the interaction between the RBD and Simeprevir/Lumacaftor, we computed the nonbonded interaction energy. GROMACS has the ability to decompose the short-range nonbonded energies via the energygrps keyword in the .mdp file. The energy terms of interest are the average short-range Coulombic interaction energy (Coul-SR) and the short-range Lennard–Jones energy (LJ-SR). The total interaction energy (IE_Binding_) is defined by:

$${\text{IE}}_{{{\text{Binding}}}} = {\text{ Coul}} - {\text{SR }} + {\text{ LJ}} - {\text{SR}}$$

## Results

### SARS-CoV-2 S glycoprotein virtual screening

**Figure 1:**
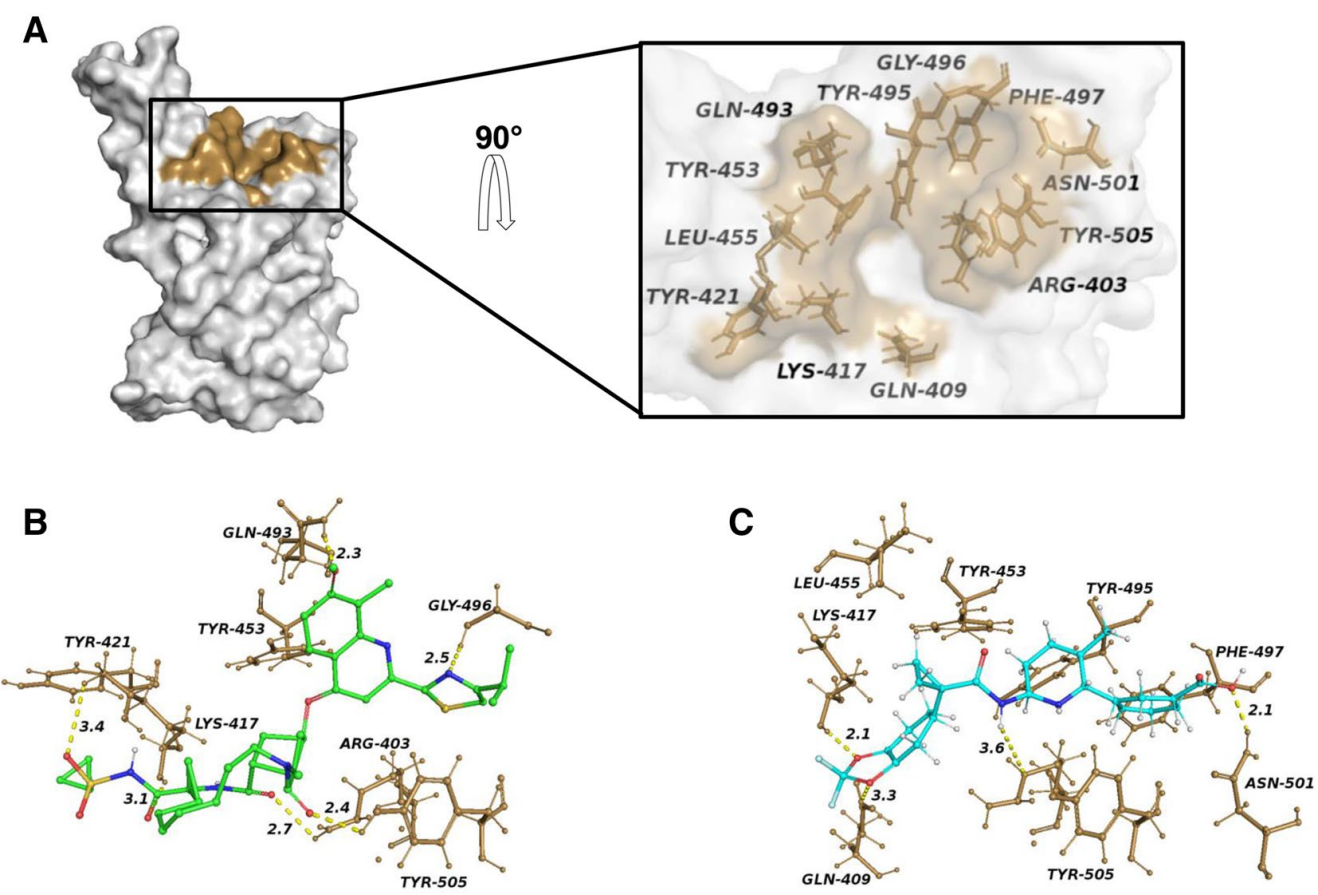
RBD binding pocket and drugs bindg site. (**A**) Surface representation of the structure of the RBD of the S protein having an open pocket conformation. The transient pocket surface patch is depicted in brown. In the zoomed region it is possible to see a detailed structural representation of the open pocket conformation. Residues laying on the pocket surface have been labelled and are shown in stick. (**B**, **C**) Structural representations of the (**B**) RBD-Simeprevir and (**C**) RBD-Lumacaftor complexes resulting from docking simulations. Residues forming direct interactions with the drugs are shown as brown sticks. Hydrogen bonds are indicated with yellow dashed lines.

SARS-CoV-2 RBD and hACE2 binding is mostly driven by polar interaction, with an overall ~ 900 Å^2^ buried surface area. A close analysis of the interface reveals the absence of cavities on RBD in the interaction surface. We performed MD simulations to account for the protein conformational flexibility and detected 1,029 transient pockets. Based on the druggability features of the cavities, i.e. volume, depth, polarity, and proximity to the hACE2 binding site, we detected a cluster of 9 transient pockets. In order to identify possible PPI inhibitors the transient pocket that contained key residues involved in hACE2 recognition and binding (Fig. 1A) was selected and used for the virtual screening of 1582 FDA-approved drugs. This curated library of drugs, whose structures were freely downloaded from DrugBank^23^, represents a reservoir of bioactive molecules that could be repurposed for COVID-19 treatment relatively fast, due to their pre-existing approval for use in humans. The 10 best compounds (Lumacaftor, Paritaprevir, Dihydroergotamine, Trypan blue, Midostaurin, Dihydroergotoxine, Simeprevir, Lurasidone, Spinosyn D, Olaparib) showed high binding free energy scores (− 9.4 to − 8.5 kcal/mol) (Fig. S1). The compound with the highest binding energy (− 9.4 kcal/mol) was Lumacaftor, a CFTR corrector that traffic the mutant protein to the plasma membrane^27^. An analysis of the quality of interactions of the 10 best compounds revealed that Simeprevir had the highest number of polar bonds with side chains of residues in the RBD binding pocket. Simeprevir, a second-generation HCV NS3/4A protease inhibitor^28^, has been reported to be both a SARS-CoV-2 main protease inhibitor^29^ and a S protein-RBD interaction inhibitor^30^. Simeprevir forms an extended network of H-bonds with Arg403, Lys417, Gln493, Gly496 and Tyr505, and forms Van Der Waals interactions with Tyr421, Tyr453 and Tyr505 (Fig. 1B). Differently, Lumacaftor has a higher number of hydrophobic contacts, compared to Simeprevir, specifically with Tyr453, Leu455, Tyr495, Phe497 and Tyr505, with the potential formation of π-stacking using the Cζ, of Arg403, and forms H-bonds with Gln409, Lys417 and Asn501 (Fig. 1C). Analysis of the crystal structure of RBD in complex with ACE2 reveals that the residues involved in the binding with the two drugs are key drivers of RBD and ACE2 interaction^6^. Of particular interest are residues Lys417, Leu455 and Gln493, which are not conserved in SARS-CoV and have been linked to the higher affinity of SARS-CoV-2 S protein for ACE2^6^. Taken together, these data show that Simeprevir and Lumacaftor are able to form clearly defined specific interactions with the SARS-CoV-2 S glycoprotein and are promising PPI competitive inhibitors.

### Simeprevir and lumacaftor inhibit RBD-ACE2 binding in silico

In order to understand if Simeprevir and Lumacaftor are able to interfere and prevent the binding between the S glycoprotein and ACE2, we ran a Supervised Molecular Dynamics (SuMD) simulations. Using SuMD it is possible to simulate the full binding process of ACE2 to RBD in presence of Simeprevir or Lumacaftor in an unbiased way (i.e. independently from starting relative positions), taking into account hydration patterns and drug binding-unbinding events. We first validated the SuMD protocol by simulating the binding process of RBD with ACE2. The resulting relative position of ACE2 bound to RBD is comparable to that in the crystal structure (Fig. S2). The interaction between ACE2 and RBD is established after 16 ns of productive trajectory and is mediated by key residues in the receptor binding motif (RBM). Specifically, SARS-CoV-2 Tyr453, Asn487, Tyr489, Gln498, Asn501 and Tyr505 form H-bonds with ACE2, whereas SARS-CoV-2 Phe486 interacts with ACE2 via van der Waals forces. Outside the RBM, we see the formation of the salt bridge between SARS-CoV-2 Lys417 and ACE2 Asp30 in line with published data suggesting that this key interaction contributes to the difference in affinity between SARS-CoV and SARS-CoV-2 S proteins for ACE2^5^. Using the same approach we then simulated the binding of ACE2 to RBD bound to Simeprevir or Lumacaftor. During the SuMD simulation ACE2 did not displace the drugs and did not form interactions with the S glycoprotein even after 50 ns of simulation. This is very likely due to the drugs interacting with the side chains of the key residues Lys417, Tyr453, Asn501 and Tyr505, which prevent ACE2 target recognition. Taken together these data show that Simeprevir and Lumacaftor prevent ACE2 recognition and binding to the S glycoprotein.

### Simeprevir and lumacaftor binding stability

**Figure 2:**
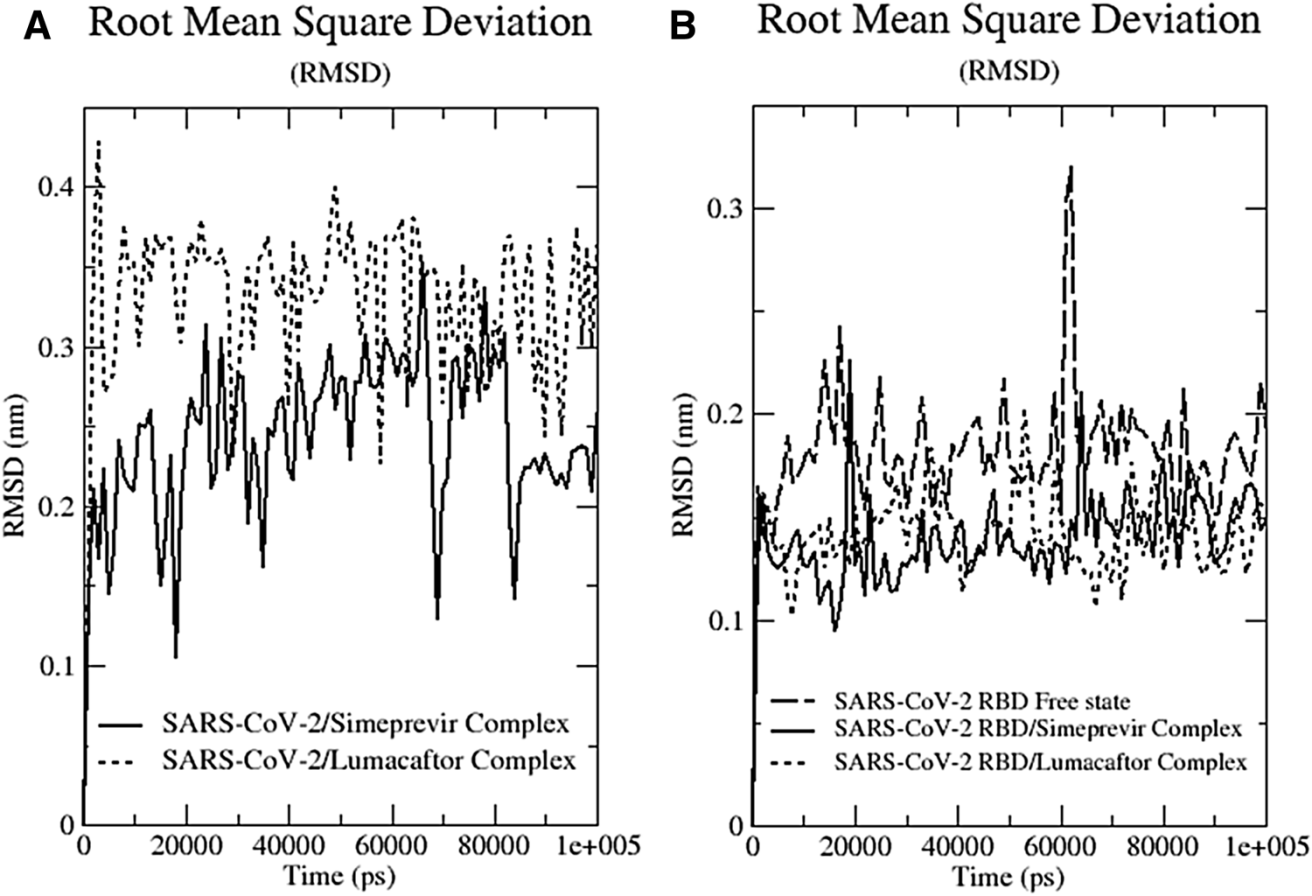
Root mean square deviation (RMSD) plots. (**A**) The RMSD profile of drugs and protein backbone, (**B**) relative to the initial frame against simulation time.

During the SuMD drugs were allowed to move and find a more energetically favourable pose in the binding pocket. We noticed very limited movements of Simeprevir and Lumacaftor and, to confirm binding stability, we performed 100 ns cMD simulations of RBD alone and in complex with the drugs. The pose of Simeprevir and Lumacaftor did not change significantly during the simulation, and the RMSD average was 2.4 Å and 3.2 Å respectively (Figs. S3 and 2A). In order to exclude presence of artefacts in our analysis, we monitored the protein structural integrity during the simulations. We noticed limited differences between the RMSD of the apo protein (1.8 Å) and the RMSD of RBD bound to Simeprevir or Lumacaftor (1.3 and 1.4 Å respectively), which excludes presence of different protein structural rearrangements in the three cMD simulations (Fig. 2B). To quantify the strength of the interaction between Simeprevir and Lumacaftor on RBD, we computed the interaction energy between the protein and the two drugs. The total interaction energy for Simeprevir and Lumacaftor was − 75.58 ± 4.2 kJ/mol and − 63.42 ± 13.8 kJ/mol respectively. Taken together these data suggest that Simeprevir and Lumacaftor bind spontaneously to the target with high affinity.

### Drugs-protein unbinding simulations

**Figure 3:**
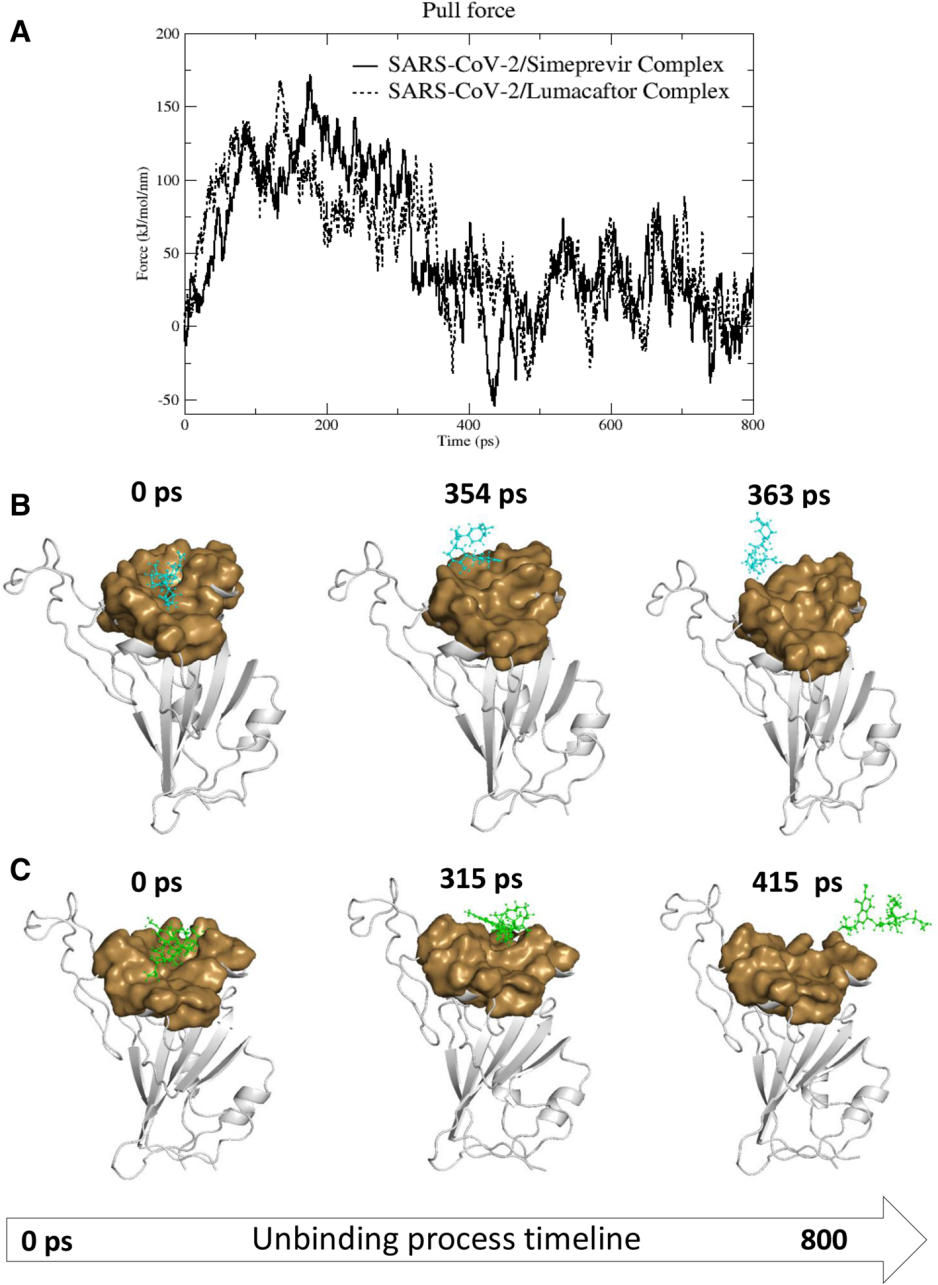
Steered Molecular Dynamics simulations. (**A**) Force profiles of drugs pulled out of the RDB transient pocket along the unbinding pathway, Lumacaftor (dotted line) and Simeprevir (continuous line). (**B**, **C**) Structural representations showing position of Lumacaftor (cyan ball-and-stick) and Simeprevir (green ball-and-stick) on RBD (white cartoon) during the different stages of the unbinding process from the RBD binding pocket (brown surface).

To further characterise the recognition process of the two drugs to the S glycoprotein we performed Steered Molecular Dynamics (SMD) simulations. We ran a 800 ps SMD simulation on RBD in complex with both Simeprevir and Lumacaftor, and the time-averaged force profiles during the unbinding simulation of complexes is shown in Fig. 3A. Both drugs have a steady increase of the applied forces on the first ~ 150 and ~ 200 ps of the simulation, respectively for Lumacaftor and Simeprevir, until they reach the maximum, which corresponds to the rupture force of Lumacaftor and Simeprevir unbinding along this dissociation pathway. The force then quickly decreases and stays constant until the end of the simulation. In the first step, between 0 and 315 ps of the simulation for Simeprevir and 0 and 354 ps for Lumacaftor, the two drugs slowly detach and move away from the transient pocket; in the second step, between 316 and 800 ps of the simulation for Simeprevir and 355 and 800 ps for Lumacaftor, they move away from the protein and enter the solvent region (Fig. 3B,C). The comparable rupture forces reflect similarity in the unbinding from RBD in line with our binding energy data.

## Discussion

SARS-CoV-2 invades human cells via ACE2, a transmembrane protein expressed on the surface of alveolar cells of the lungs. Upon binding of ACE2, viral and host cell membranes fuse and the virus enters into the host cell. This results in the development of an infectious disease, called COVID-19, which is associated with a major immune inflammatory response. Deaths are caused by respiratory failure, which have been linked to a cytokine storm with high serum levels of pro-inflammatory cytokines and chemokines^31^. There are currently no approved vaccines or drugs that specifically target Coronavirus infection, and, despite several ongoing clinical trials, treatment options have been based on different clinical approaches with limited background testing. An exponentially growing number of computational studies have tried to provide molecular data in support of these novel potential COVID-19 treatments^15, 32–34^.

The aim of this proof of principle study was to propose a robust in silico protocol that overcame limitations of classic virtual screening studies^35^. The role of hydration patterns in target recognition and binding is completely absent in docking simulations. Furthermore, in most virtual screenings, while the ligand is flexible, proteins are only semi-flexible, which affects both the resulting pose of the ligand and the scoring system^36^. More reliable information can only be obtained by MD simulations, which, despite being computationally expensive, allow to take into account macromolecules’ unique features, such as conformational flexibility, charge distribution, and hydration patterns in target recognition, drug binding, and drug unbinding^37,38^. In this study we coupled docking with cMD, SuMD and SMD to identify a Spike protein—ACE2 interaction inhibitor. Transmission electron microscope image of SARS-CoV-2 have shown how the viral envelope is densely populated by the S protein, which, due to its role in pathogenesis, is the main target of neutralizing antibodies and vaccines^39^. An analysis of the crystal structure of the RBD with ACE2 reveals that the RBD of the S protein has a relatively flat surface, which would be unsuitable for drug targeting. Previous studies have shown that the analysis of protein dynamics allows for the identification of transient pockets where small molecules can bind proteins^40^. We identified a transient pocket with druggability features on the RBD which may represent a hot spot^38^. Indeed, comparison with the structure of SARS-CoV S protein in complex with a neutralising antibody isolated from a SARS-CoV survivor shows that the pocket we identified lies on the same surface recognised by the CDRs of the antibody^39^. We retrieved the structure of the protein with an open pocket from the trajectory of the MD simulation and we used it for a virtual screening of 1582 FDA-approved drugs. The advantage of focusing on FDA-approved drugs is that the safety issues are all within suitable bounds and are well understood, meaning that they could proceed to clinical trial reasonably quickly. The compounds showing high binding energies and forming a network of specific interaction with side chains of residues in the RBD binding pocket were Simeprevir and Lumacaftor. Simeprevir, a direct-acting antiviral agent for the treatment of HCV infections, is a second generation of orally available NS3/4 HCV protease inhibitor^41^. Lumacaftor is a CFTR corrector that stabilises the first transmembrane domain of CFTR, resulting in an improved maturation of CFTR mutants^42^. The two drugs were also selected for their reported minimal off-targeting, suggesting a lack of binding to other human proteins^41,43^. Furthermore, Kadioglu et al.^44^ also identified Simeprevir as a potential S protein-ACE2 interaction inhibitor. In a similar attempt to overcome limitations of classic in silico docking, they adopted a complex approach combining virtual drug screening, molecular docking and supervised machine learning techniques^44^. While their results strongly reinforce the findings of our study, our strategy provided comprehensive information about the ability of compounds to interfere with the Spike protein binding to ACE2 by combining cMD, SuMD and SMD.

Virtual screening and in vitro studies suggested that Lumacaftor and Simeprevir are also SARS-CoV-2 main protease inhibitors^45^. Interestingly, several in silico and in vitro studies have identified antiviral agents targeting HCV infection (single-stranded negative-sense RNA virus) as promising treatments for COVID-19^46^, which include HCV approved inhibitors of the viral RNA synthesis, the 3CL protease and the helicase activity^46^. Antiviral agents against HCV infections have also been studied for their promising ability to interfere with other viral infections caused by RNA viruses, i.e. SARS-associated coronavirus^47^, MERS^48^, Enterovirus A71, Herpes simplex virus type 1 and Zika virus^41^. This would suggest the possibility to use and/or develop Simeprevir into broad-spectrum antiviral drugs^41^. Simeprevir and Lumacaftor are also promising for their potential ability to inhibit multiple steps of the SARS-CoV-2 infection by interfering with the S protein binding to the ACE2 receptor and by inhibiting the SARS-CoV-2 main protease, essential for processing the polyproteins that are translated from the viral RNA^49^. The concept of multi-target drugs that inhibit several proteins simultaneously has been successfully used for the treatment of many diseases. For example, the anti-HIV drug Cosalane was developed to inhibit binding of the HIV gp120 envelope glycoprotein to CD4 and simultaneously to inhibit the cytopathic mechanism of HIV-1^50^.

While writing this paper, several drug repurposing studies targeting the S protein have been published. Interestingly, several papers^32,34,44,51^ carried out virtual screenings on the same surface we identified as a transient pocket. Binding energies of proposed compounds are however lower than the one we observed for Simeprevir and Lumacaftor. This is very likely linked to the protein structures used for virtual screening and/or a binding pocket not being in the optimal open conformation, highlighting the strength of our in silico approach.

Our results show the importance of taking into account the full structural features of a protein–ligand complex and how a combination of MD simulations may help predict the validity of a proposed inhibitor. Our work suggests that Simeprevir and Lumacaftor could be potential initial compounds able to prevent and treat SARS-CoV-2 infection.

## Supplementary information

Supplementary Information.
