# Supplementary material for: An integrated drug repurposing strategy for the rapid identification of potential SARS-CoV-2 viral inhibitors
Source: Sci Rep. 2020 Aug 17;10:13866. doi: 10.1038/s41598-020-70863-9 (PMC7431416; doi:10.1038/s41598-020-70863-9)

An integrated drug repurposing strategy for the rapid identification of potential SARS-CoV-2 viral inhibitors.

Alfonso Trezza[1#], Daniele Iovinelli[1#], Annalisa Santucci[1], Filippo Prischi[2*] and OttaviaSpiga[2*]

[1]Department of Biotechnology, Chemistry and Pharmacy, University of Siena, 53100, Siena, Italy
[2]School of Life Sciences, University of Essex, Colchester, CO4 3SQ, UK

alfonso.trezza2@unisi.it
daniele.iovinelli@student.unisi.it
annalisa.santucci@unisi.it
fprischi@essex.ac.uk
ottavia.spiga@unisi.it

Figure S1. VINA Docking output summary. Top 10 scoring FDA approved drugs for the RBD of the S protein, with reported docking binding energies and toxicity values.

Figure S2. Supervised Molecular Dynamics Simulation. Structural representation of the SuMD simulation compared to the reference crystal structure. The reference RBD-ACE2 complex structure (PDB 6LZG) is shown with a cartoon and surface representation, with the RBD of the S protein in cyan bound to ACE2 in orange. At the beginning of the SuMD simulations the two protein are positioned far from each other, with the RBD shown in white, (A) and at the end of the simulation the proteins adopt the same relative orientation as in the reference structure (B). The structures have been aligned using the ACE2.

Figure S3. Drugs optimised pose after MD. Structural comparison of drug poses at the beginning (docking pose) and at the end of the 100 ns cMD. (A) Simeprevir docking structure is shown in green ball-and-stick and the structure at the end of the simulation is shown in light green. (B) Lumacaftor docking structure is shown in cyan ball-and-stick and the structure at the end of the simulation is shown in light cyan.

Figure S1

Ligand	ΔG (Kcal/mol)	Toxicity
Lumacaftor	-9.4	4/6
Paritaprevir	-9.3	3/6
Dihydroergotamine	-9.2	4/6
Trypan blue	-9.1	6/6
Midostaurin	-8.9	4/6
Dihydroergotoxine	-8.9	4/6
Simeprevir	-8.7	4/6
Lurasidone	-8.6	4/6
Spinosyn D	-8.6	1/6
Olaparib	-8.5	4/6

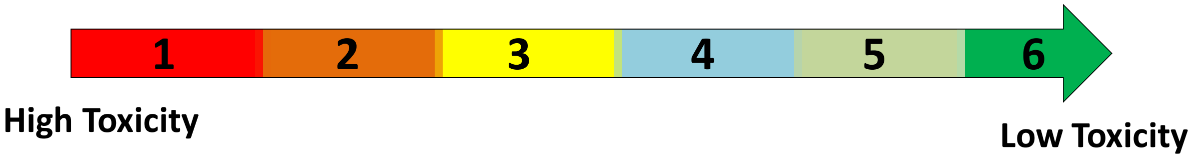

Figure S2

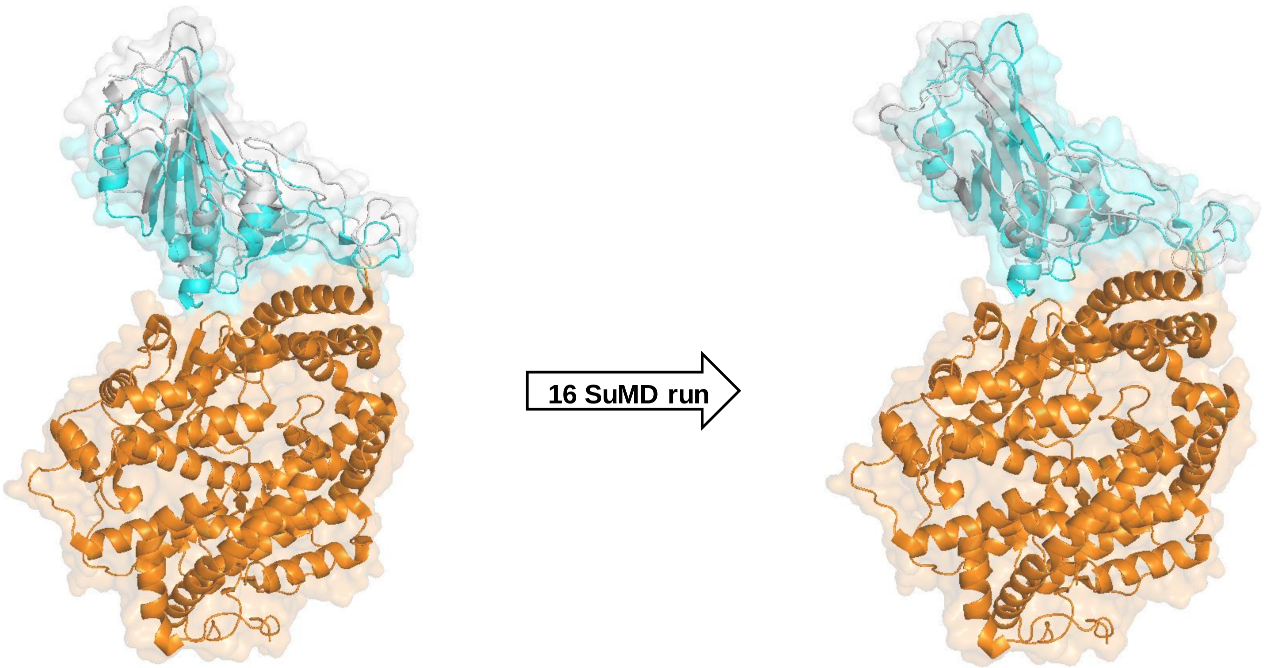

Figure S3

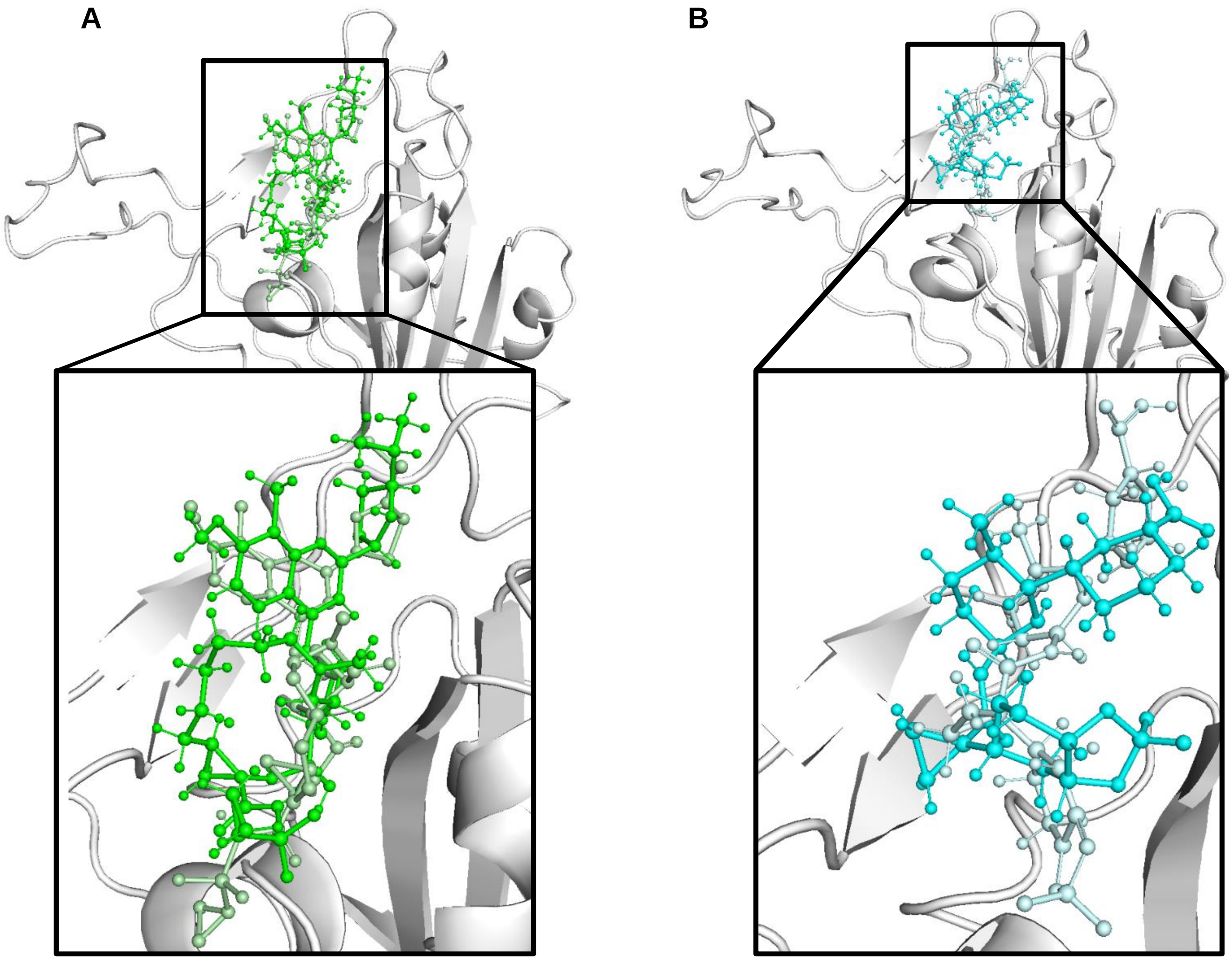

Supplement: Supplementary file 1 — Supplementary Information. [file 41598_2020_70863_MOESM1_ESM.pdf]
